# Posterior Hip Pointer: Subperiosteal Detachment of the Gluteal Muscles at the Posterior Iliac Crest in Two Elite Athletes

**DOI:** 10.3390/muscles4020012

**Published:** 2025-04-23

**Authors:** Joffrey Drigny, Amélie Labrousse, Marion Remilly, Emmanuel Reboursière

**Affiliations:** 1 Service de Médecine Physique et de Réadaptation, Service de Médecine du Sport, CHU de Caen Normandie, Normandie Univ, UNICAEN, INSERM, COMETE, GIP CYCERON, 14000 Caen, France; 2 Service de Médecine du Sport, CHU de Caen Normandie, Normandie Univ, UNICAEN, 14000 Caen, France

**Keywords:** hip injuries, iliac crest, athletic injuries, ultrasonography

## Abstract

Hip injuries are common in contact sports, particularly in high-impact activities. A well-known type of hip trauma is the hip pointer, which is a contusion of the iliac crest caused by a direct blow. Typically, hip pointers involve the lateral aspect of the iliac crest. In this case report, we present an unusual variation of this injury affecting the posterior iliac crest near the posterior superior iliac spine (PSIS). We describe two cases of elite athletes who sustained posterior iliac crest trauma, a condition we propose naming the “posterior hip pointer”. This report highlights the clinical presentation, imaging findings, treatment approach, and implications for sports medicine.

## 1. Introduction

Hip injuries are frequent in contact sports, where athletes are exposed to high-velocity collisions, falls, and direct impacts [1]. These injuries range from soft tissue contusions and muscular strains to fractures and avulsions, significantly affecting an athlete’s performance and recovery timeline. Most hip pain cases are either anterior or lateral, but posterior hip pain, although relatively uncommon, is gaining more attention within the sports medicine community [2]. Patients’ complaints are often vague or nonspecific, and the pain is frequently caused by factors outside the hip joint, making both diagnosis and treatment decisions more challenging.

One of the most commonly recognized injuries of the hip region is the hip pointer, a term first introduced by Blazina in 1967 to describe a direct contusion of the iliac crest [3]. The classic hip pointer is caused by a direct blow to the iliac crest, often occurring during tackles or falls onto a hard surface. Unlike muscular strains or avulsion fractures, which typically result from rotational or twisting forces, hip pointers arise exclusively from blunt force trauma. Nevertheless, the associated soft tissue can include subperiosteal edema, bleeding from the nutrient vessels of the bone, or hematoma formation within the surrounding musculature, all of which contribute to localized pain and dysfunction [4].

Hip pointers are a common injury, especially in male athletes participating in contact sports, and are considered one of the most frequent causes of hip injuries among collegiate athletes [1]. Traditionally, hip pointers are localized to the lateral aspect of the hip, where the iliac crest is most exposed to impact [5]. The injury mechanisms may involve the compression of the abductor muscles against the iliac crest, leading to significant pain and movement restrictions. Due to their prevalence in contact sports, hip pointers are well recognized in sports medicine [6].

Despite the well-established nature of classic hip pointers involving the lateral aspect of the iliac crest, trauma involving the posterior iliac crest is rarely documented. In this case report, we present two elite athletes who sustained impact injuries to the posterior iliac crest near the posterior superior iliac spine (PSIS). Due to the similarities in the mechanism and presentation with traditional hip pointers, we propose the term “posterior hip pointer” to describe this specific variant.

## 2. Detailed Case Description

### 2.1. Case Presentation

- Case 1: A handball player with posterior pelvis trauma

A 16-year-old male elite handball player sustained two falls onto his lower back, once during training and once during match play, over a 5-day interval. He reported localized pain and tenderness over the posterior iliac crest near the PSIS, with mild swelling and discomfort during hip movements. A clinical examination revealed tenderness over the posterior iliac crest, mild functional impairment, and an avoidance of direct contact due to pain.

- Case 2: An ice hockey player with posterior pelvis trauma

**Figure 1:**
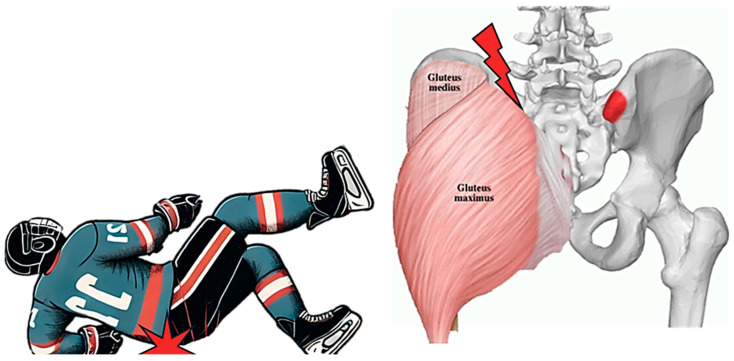
The mechanism of injury and pain location in a posterior hip pointer due to a backward fall in a hockey player.

A 20-year-old male elite ice hockey player experienced a similar fall onto the ice, landing forcefully on his lower back (Figure 1). He reported pain in the posterior iliac crest, localized swelling, and difficulty tolerating direct pressure. Like Case 1, he avoided contact due to the discomfort but maintained relatively normal movement patterns aside from pain-related limitations.

### 2.2. Imaging Findings

**Figure 2:**
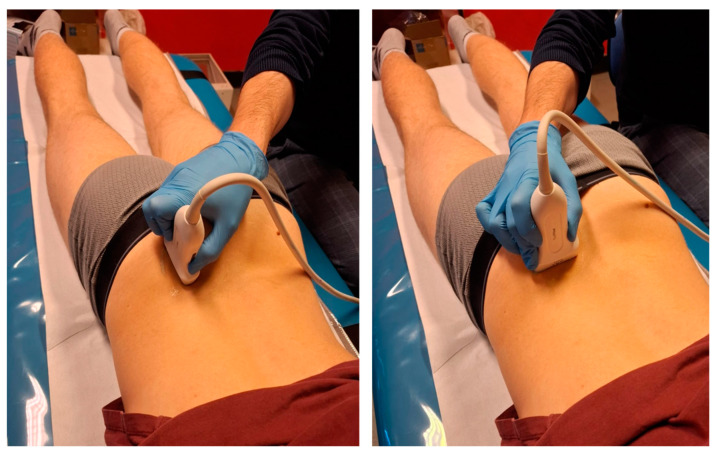
Representation of transducer positioning for (**left**) sagittal plane and (**right**) transverse plane ultrasound scans of posterior iliac crest.

**Figure 3:**
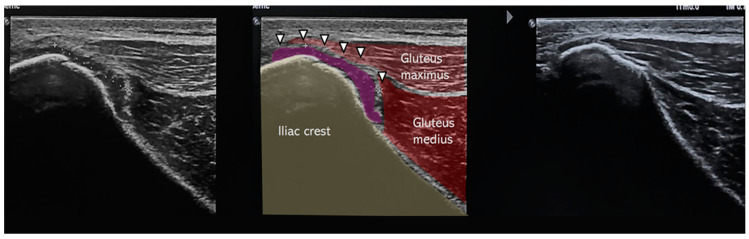
Sagittal ultrasound scans of posterior iliac crest in Case 1: (**left**) injured side, (**middle**) annotated scan highlighting periosteal detachment (arrowhead), and (**right**) uninjured side for comparison.

**Figure 4:**
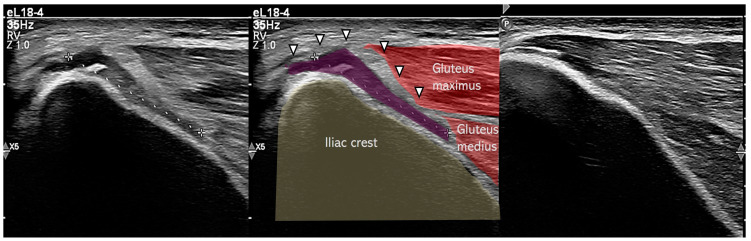
Sagittal ultrasound scans of posterior iliac crest in Case 2: (**left**) injured side, (**middle**) annotated scan highlighting periosteal detachment (arrowhead), and (**right**) uninjured side for comparison.

One week after the injury, both athletes underwent ultrasound imaging (Philips EPIQ 7, eL18-4 transducer) performed by an experienced sports medicine physician (Figure 2, Figure 3 and Figure 4).

The findings were strikingly similar and included the following:

- Subperiosteal hematoma at the posterior iliac crest;
- A 20–25 mm detachment of the gluteus maximus;
- Partial involvement of the gluteus medius.

Despite the presence of significant hematoma formations, no hematoma drainage was performed, as both athletes exhibited progressive improvement.

### 2.3. Treatment and Outcome

Both athletes were treated with conservative management, including a short period of contact avoidance, the use of analgesic medication (initially acetaminophen and secondary non-steroidal anti-inflammatory drugs as needed), and a progressive return to sport with the use of padded protective wear.

The use of padded technical suits facilitated full sports participation within two weeks, minimizing the impact of the injury on their season.

**Figure 5:**
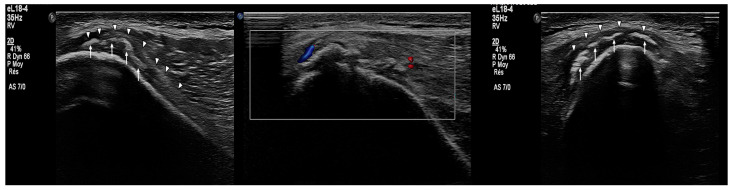
The three-week follow-up ultrasound scans of the posterior iliac crest after the recurrent falls in Case 2: (**left**) the sagittal plane in B-mode alone, (**middle**) combined with color Doppler, and (**right**) the transverse plane, showing periosteal thickening (arrowhead) and calcification (arrow).

Three weeks after the initial injury, Case 2 (the ice hockey player) experienced contact with an opponent on his posterior hip during a game, leading to a recurrence of pain. The ultrasound imaging revealed calcification formations within the subperiosteal detachment along with mild hyperemia on the Doppler imaging, suggesting some ongoing inflammation (Figure 5). However, there was no evidence of worsening of the previously injured structures.

**Figure 6:**
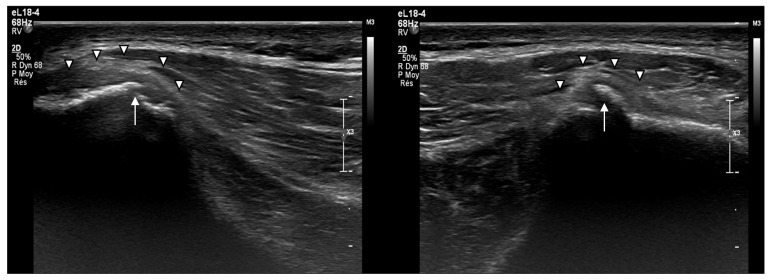
The seven-month follow-up ultrasound scans of the posterior iliac crest in Case 1: (**left**) the sagittal plane and (**right**) the transverse plane, showing the periosteal thickening (arrowhead) and calcification (arrow).

A routine seven-month follow-up evaluation for Case 1 (the handball player) revealed persistent cortical irregularity, lamellar calcifications, and periosteal thickening indicative of bone remodeling after trauma (Figure 6). However, the athlete remained completely asymptomatic, suggesting that these sequelae may be common yet clinically insignificant in similar cases.

## 3. Discussion

In addition to the classic hip pointer, which typically affects the lateral aspect of the iliac crest due to a direct blow, we describe a posterior variant, termed the “posterior hip pointer”, affecting the posterior iliac crest near the PSIS. While the mechanism of injury remains a direct impact, the mechanism of injury and the anatomical location differ significantly, involving structures such as the gluteus maximus (Table 1). Additionally, this study provides valuable insights into the soft tissue and bone remodeling during follow-up.

In handball—a sport defined by rapid directional changes, jumping, and frequent physical contact—pelvic and hip injuries constitute a significant share of the musculoskeletal complaints, especially among elite athletes [7]. While less common than knee or ankle injuries, they still represent a notable portion of bone-related injuries. Similarly, in ice hockey, where high-speed skating, body checks, and falls onto the ice create a high-risk environment, hip injuries are prevalent [8,9]. Traditional hip pointers are among the most common and painful pelvic/hip injuries in this sport, caused by a hard check crushing the hip abductor muscles against the iliac wing [10].

According to Frank et al., the most frequent causes of posterior hip pain in athletes include sacroiliac joint dysfunction, hip extensor or rotator strain, proximal hamstring rupture or avulsion, and piriformis syndrome or referred pain from the lumbar spine, particularly in chronic cases [2]. We present here a potentially under-recognized cause of traumatic posterior hip pain, highlighting the need for further studies to evaluate its frequency and impact. Understanding less recognized causes of posterior hip pain could enhance the diagnosis and treatment strategies for athletes experiencing persistent or unexplained hip pain.

Some variants of lateral hip pointers have been described, including an internal degloving and avulsion injury to the internal oblique insertion on the iliac crest in Australian football players [11] and hockey players [12]. To our knowledge, this is the first report describing posterior hip pointers with strikingly similar characteristics in two young elite athletes. Both cases involved direct trauma to the lower back/pelvis, leading to pain and discomfort, localized swelling, and tenderness over the posterior iliac crest near the PSIS. According to Frank et al., the primary causes of posterior hip pain in athletes often include referred pain from the lumbar spine, sacroiliac joint dysfunction, a hip extensor or rotator muscle strain, and a proximal hamstring rupture [2]. Physical examinations helped rule out these possibilities, supporting the hypothesis of a posterior iliac crest contusion.

Ultrasound imaging proved crucial for both the diagnosis and follow-up. The key findings included the following:

- Subperiosteal hematoma formation at the posterior iliac crest;
- Partial detachment of the gluteus maximus (~20–25 mm), with involvement of the gluteus medius;
- Persistent cortical irregularities, including calcifications and periosteal thickening, on long-term follow-up.

The presence of calcification formations in Case 2, detected on ultrasound following a second impact at three weeks, suggests early bone remodeling and healing processes. Additionally, mild hyperemia on the Doppler imaging indicated a low-grade inflammatory response, but without evidence of worsening injury.

Although not used in our cases, MRI can add diagnostic specificity in complex or equivocal cases of hip pointers by providing more detailed imaging than ultrasound. While ultrasound is useful for assessing soft tissue and superficial injuries, MRI offers superior visualization of bone marrow edema (e.g., bone bruise) and trabecular microfractures, which are characteristic of hip pointers [13]. Additionally, MRI can reveal surrounding soft tissue edema and better assess the severity and extent of the injury, which might be missed or unclear on ultrasound alone [14]. Muscle injury classifications and grading systems on both ultrasound and MRI can aid in standardizing the findings and improving the diagnostic consistency and comparability [15]. While MRI is particularly valuable in cases of diagnostic uncertainty or complexity, its higher cost and limited availability compared to ultrasound should be considered. Unlike MRI, ultrasound can perform dynamic assessments and guide aspiration, making it a more practical and cost-effective option in certain cases [16].

Given that hip pointers are primarily managed conservatively, treatment for these cases followed a similar approach, including contact avoidance, local cryotherapy, analgesic medication for symptom control, and a progressive return to sport within two weeks using protective padding. In the early stage, nonsteroidal anti-inflammatory drugs were avoided to reduce the risk of increased bleeding and a larger hematoma. The treatments aligned with the standards for treating hip pointers, especially in ice hockey players [10]. In the case of a large hematoma, it has been suggested that aspiration can help alleviate pain and may reduce the risk of developing myositis ossificans [17]. However, there is no definitive evidence supporting the benefits of aspiration for small hematomas, nor are there specific guidelines on the minimum volume required to justify the procedure. A retrospective analysis of rugby players found that local anesthetic injections were safe and may facilitate the resumption of sporting activities after an iliac crest contusion [18]. The rapid return to play in both cases, aided by padded protective suits, suggests that the conservative treatment is effective for posterior hip pointers, as long as no severe structural damage is present. Although conservative management is the preferred approach, more aggressive interventions, such as surgical repair, may be considered in cases involving severe structural damage or large muscle avulsions. Indeed, in rare cases, Lohrer et al. have described variants of hip pointers, including large muscle avulsions, which have been treated surgically by refixation of the aponeurotic obliquus internus muscle to the lateral iliac crest [19].

In our case, the athletes could return to sport in two weeks without significant impairment or the need for specific rehabilitation, but in more severe cases, a longer recovery and targeted rehabilitation would be necessary to ensure full readiness. Interestingly, LaPrade et al. suggested that a hip abduction exercise rehabilitation program may help athletes regain strength after classic hip pointers, especially in ice hockey players [10]. The rehabilitation strategies should prioritize pain management and mobility exercises, followed by progressive loading, with a particular emphasis on eccentric exercises to support the healing process [20]. Additionally, targeted core exercises, gluteus maximus strengthening, and sport-specific drills should be incorporated to enhance stability and functional performance and to ensure a safe and effective return to play. Functional performance tests, including hop tests, agility drills, and sport-specific movements, are essential for evaluating an athlete’s strength, power, stability, and agility to ensure their readiness to return to sport [21]. Additionally, assessments such as the single-leg stance test, deep squat test, squat test, and star excursion balance test (SEBT) have demonstrated validity for use in individuals with suspected hip dysfunction [22]. Furthermore, isokinetic testing of the hip muscles can provide valuable insights into muscle strength and symmetry after an injury, especially for hip extensors [23]. These tests help identify deficits in neuromuscular control, limb symmetry, and movement strategies, ensuring a safe transition from rehabilitation to full participation in the sport.

With regard to this case report, we are unable to identify specific pelvic morphology that is at risk for such injuries, although all athletes may be at risk, particularly due to the vulnerable nature of the iliac crest. It is more likely that athletes with low subcutaneous fat would be more exposed to such blunt traumas, but further investigations could focus on pelvic morphology, such as the pelvic tilt, as a risk factor for posterior hip pointers [24]. Special attention should be given to younger athletes, as apophyseal avulsion fractures of the pelvis and hip are common in this age group [25]. In most cases, these fractures result from sudden, forceful muscle contractions or passive lengthening during activities like acceleration, jumping, or kicking; less frequently, they occur due to direct trauma [26]. They most often occur at the anterior inferior iliac spine (AIIS), followed by the anterior superior iliac spine (ASIS) and the ischial tuberosity. Less commonly, fractures affect the iliac crest, with posterior iliac crest injuries being rare. Beyond the morphological parameters, additional risk factors, such as equipment or suits, surface hardness, and the athlete’s position during a fall, especially hip flexion, should be thoroughly evaluated and further investigated in relation to this type of trauma. Research has shown that the mechanisms of hip injuries differ across sports and between genders [1]. Men are more likely to experience contact-related injuries, while women are more prone to overuse injuries. Therefore, studies specifically exploring the impact of individual and external factors on these injuries are needed.

This case report is limited by the small sample size (n = 2) and the lack of long-term functional outcome assessments beyond the imaging findings. Future studies should aim to establish standardized diagnostic criteria for posterior hip pointers and confirm their distinction from lateral hip pointers and other iliac crest injuries. Additionally, research should evaluate the role of imaging modalities, particularly MRI, in detecting deeper soft tissue involvement and potential long-term sequelae. Further investigation is also needed to assess the efficacy of various protective strategies (such as padded sportswear), to determine the optimal treatment approach for a safe and timely return to sport, and to explore the long-term functional outcomes. Finally, given the lack of similar detailed descriptions, the specific incidence of this injury remains uncertain, making it unclear whether it is genuinely rare or simply under-recognized. However, due to the frequency of situations that may induce such blunt trauma, this injury pattern is more likely under-recognized rather than rare, as diagnostic limitations and overlap with other musculoskeletal conditions may contribute to its underreporting in clinical practice.

## 4. Conclusions

This case report introduces the term “posterior hip pointer” to describe trauma affecting the posterior aspect of the iliac crest, which is characterized by a subperiosteal hematoma and muscle detachment, with a periosteum reaction and bone remodeling in the long term. Our findings indicate that ultrasound imaging is valuable for the diagnosis and monitoring, while conservative management with protective padding and analgesia effectively supports a timely return to sport. While this case report is based on only two patients, these findings offer valuable insights and provide a foundation for further research to assess their broader applicability.

## Figures and Tables

**Table 1:** Comparison of classic hip pointer [4] and posterior hip pointer injuries.

Feature	Classic Hip Pointer	Posterior Hip Pointer
Location	Lateral aspect of the iliac crest	Posterior iliac crest near the PSIS
Mechanism of Injury	Direct impact to the iliac crest; Contact (out-of-bounds object/person-opponent)	Direct impact to the lower back/pelvis; Contact (floor/surface)
Involved Structures	Gluteus medius, tensor fasciae latae, anterolateral abdominal wall (obliquus and transversus abdominis)	Gluteus maximus, gluteus medius
Symptoms	Localized pain, swelling, tenderness over lateral iliac crest	Localized pain, swelling, tenderness over posterior iliac crest
Imaging	Ultrasound, MRI, X-ray (if fracture/apophyseal injuries suspected)	Ultrasound, MRI (if fracture suspected, apophyseal injuries less common)
Treatment	Contact avoidance Compression and icing to reduce swelling Pain management (avoiding NSAIDs) Combination of local anesthetic and corticosteroid for pain relief (to be considered) Rehabilitation focusing on hip range of motion and hip abduction/abdominal wall strengthening	Similar treatment strategies Rehabilitation focusing on hip abduction/extension, powerful movements (upward/forward propulsion)
Complications	Rare cases of bone exostosis or periostitis Possible temporary sensory nerve block Uncommon bruising extending to the lower body	Persistent cortical irregularities, calcifications, periosteal thickening

PSIS: posterior superior iliac spine; MRI: magnetic resonance imaging; NSAIDs: non-steroidal anti-inflammatory drugs.

## Data Availability

The data presented in this study are available on request from the corresponding author.

## Abbreviations

The following abbreviations are used in this manuscript:

PSIS	posterior superior iliac spine
SEBT	star excursion balance test
AIIS	anterior inferior iliac spine
ASIS	anterior superior iliac spine
MRI	magnetic resonance imaging
NSAIDs	non-steroidal anti-inflammatory drugs

## References

[1] Kerbel Y.E., Smith C.M., Prodromo J.P., Nzeogu M.I., Mulcahey M.K. (2018). Epidemiology of Hip and Groin Injuries in Collegiate Athletes in the United States. Orthop. J. Sports Med..

[2] Frank R.M., Slabaugh M.A., Grumet R.C., Virkus W.W., Bush-Joseph C.A., Nho S.J. (2010). Posterior Hip Pain in an Athletic Population: Differential Diagnosis and Treatment Options. Sports Health.

[3] Blazina M.E. (1967). The “Hip-Pointer”, a Term to Describe a Specific Kind of Athletic Injury. Calif. Med..

[4] Hall M., Anderson J. (2013). Hip Pointers. Clin. Sports Med..

[5] Grumet R.C., Frank R.M., Slabaugh M.A., Virkus W.W., Bush-Joseph C.A., Nho S.J. (2010). Lateral Hip Pain in an Athletic Population. Sports Health.

[6] Anderson K., Strickland S.M., Warren R. (2001). Hip and Groin Injuries in Athletes. Am. J. Sports Med..

[7] Martín-Guzón I., Muñoz A., Lorenzo-Calvo J., Muriarte D., Marquina M., de la Rubia A. (2021). Injury Prevalence of the Lower Limbs in Handball Players: A Systematic Review. Int. J. Environ. Res. Public Health.

[8] Wörner T., Thorborg K., Clarsen B., Eek F. (2022). Incidence, Prevalence, and Severity of and Risk Factors for Hip and Groin Problems in Swedish Male Ice Hockey Players: A 1-Season Prospective Cohort Study. J. Athl. Train..

[9] Kuhn A.W., Noonan B.C., Kelly B.T., Larson C.M., Bedi A. (2016). The Hip in Ice Hockey: A Current Concepts Review. Arthrosc. J. Arthrosc. Relat. Surg..

[10] LaPrade R.F., Wijdicks C.A., Griffith C.J. (2009). Division I Intercollegiate Ice Hockey Team Coverage. Br. J. Sports Med..

[11] Gultekin S., Cross T. (2019). The Franklin-Naismith Lesion: A Severe Variant of Hip Pointer. Orthop. J. Sports Med..

[12] Narciso R., Venis L., Cardoos N. (2024). Distal Abdominal Oblique Avulsion Injuries in Two Collegiate Hockey Players: A Case Report. Curr. Sports Med. Rep..

[13] Ladd L.M., Blankenbaker D.G., Davis K.W., Keene J.S. (2014). MRI of the Hip: Important Injuries of the Adult Athlete. Curr. Radiol. Rep..

[14] Hegazi T.M., Belair J.A., McCarthy E.J., Roedl J.B., Morrison W.B. (2016). Sports Injuries about the Hip: What the Radiologist Should Know. Radiogr. Rev. Publ. Radiol. Soc. N. Am. Inc.

[15] Grassi A., Quaglia A., Canata G.L., Zaffagnini S. (2016). An Update on the Grading of Muscle Injuries: A Narrative Review from Clinical to Comprehensive Systems. Joints.

[16] Orlandi D., Corazza A., Arcidiacono A., Messina C., Serafini G., Sconfienza L.M., Silvestri E. (2016). Ultrasound-Guided Procedures to Treat Sport-Related Muscle Injuries. Br. J. Radiol..

[17] Varacallo M.A., Bordoni B. (2025). Hip Pointer Injuries. StatPearls.

[18] Orchard J.W., Steet E., Massey A., Dan S., Gardiner B., Ibrahim A. (2010). Long-Term Safety of Using Local Anesthetic Injections in Professional Rugby League. Am. J. Sports Med..

[19] Lohrer H., Höferlin A. (2023). Successful Repair of M. Obliquus Internus Abdominis Avulsion at the Iliac Crest-Operative Technique in Professional Soccer Players. Orthop. Surg..

[20] Notarnicola A., Ladisa I., Lanzilotta P., Bizzoca D., Covelli I., Bianchi F.P., Maccagnano G., Farì G., Moretti B. (2023). Shock Waves and Therapeutic Exercise in Greater Trochanteric Pain Syndrome: A Prospective Randomized Clinical Trial with Cross-Over. J. Pers. Med..

[21] Draovitch P., Maschi R.A., Hettler J. (2012). Return to Sport Following Hip Injury. Curr. Rev. Musculoskelet. Med..

[22] Kivlan B.R., Martin R.L. (2012). Functional Performance Testing of the Hip in Athletes: A Systematic Review for Reliability and Validity. Int. J. Sports Phys. Ther..

[23] Julia M., Dupeyron A., Laffont I., Parisaux J.-M., Lemoine F., Bousquet P.-J., Hérisson C. (2010). Reproducibility of Isokinetic Peak Torque Assessments of the Hip Flexor and Extensor Muscles. Ann. Phys. Rehabil. Med..

[24] Suits W.H. (2021). Clinical Measures of Pelvic Tilt in Physical Therapy. Int. J. Sports Phys. Ther..

[25] Sinha R., Johnson B., Morris W.Z., Wilson P.L., Ellis H.B. (2024). Hip Injuries in the Pediatric Athlete—Pelvic Apophyseal Avulsions. Oper. Tech. Sports Med..

[26] Filippo C., Alessandro N., Cristina G., Margherita M., Francesco P., Francesco C. (2018). Apophyseal Avulsion Fractures of the Pelvis. A Review. Acta Biomed. Atenei Parm..

